# Early onset breast cancer in black British women: a letter to the editor of *British Journal of Cancer* regarding early onset of breast cancer in a group of British black women

**DOI:** 10.1038/sj.bjc.6604336

**Published:** 2008-04-15

**Authors:** S Dindyal, M J Ramdass, V Naraynsingh

**Affiliations:** 1 Royal Cornwall Hospital, General Surgery Department Truro, Cornwall TR1 3LJ, UK; 2 General Hospital, Port-of-Spain, Trinidad, West Indies

**Sir**,

I was delighted to read your recent electronic publication of the study concerning early onset of breast cancer in a group of British black women by Bowen *et al* (2008) from Barts and The London/Homerton Hospital. In 2002, I carried out a similar retrospective analysis of 2 years of breast cancer data concerning 299 patients treated at The General Hospital, Port-of-Spain, Trinidad (Dindyal *et al*, 2004).

Trinidad, an island in the southern most part of the Caribbean Sea, adjacent to South America, has a population with a diverse racial mixture, similar to Hackney. The principle ethnic groups in Trinidad are African (43%) and Eastern Indian (41%), which account for most of the population. These two ethnic groups are fourth generations of slaves brought to Trinidad in the late 1800s to work in the plantations. Breast cancer is the most prevalent carcinoma amongst women in Trinidad.

We found that Afro-Caribbeans were the most commonly affected race (54%) followed by Indo-Caribbeans (35%) then the mixed-race group (11%). The average age of the patients and ranges are as follows: Afro-Caribbean average age 53 years (range 28–89 years), Indo-Caribbean average age 55 years (range 29–76 years) and mixed race average age 59 years (range 29–80 years).

Our study, similar to study of Bowen *et al* on blacks and whites, showed that Afro-Caribbeans were almost twice as likely to develop intraductal or intralobular carcinoma compared to the Indian-Caribbean population of Trinidad. We found no differences between our groups with respect to age of presentation as Bowen *et al*.

We also analysed benign breast lumps between the same ethnic groups and found no significant differences but confirmed that fiboadenoma is the commonest benign breast lump, which occurs principally in young women (Dindyal *et al*, 2007).

It is well documented in the literature, mostly from United States, that African-American women have a worse breast cancer survival rate and a significantly younger age of diagnosis, such as in a large study of 135 000 women by Joslyn and West (2000).

The questions that I would like to raise are regarding clarification of the heritage of the black women included in this study. Were they African or West Indian, and were the white women born in England or were they Jewish or Eastern European. Were mixed-race women included in the study or excluded. The importance of original heritage is highlighted in a study by Harris (1977), which showed that breast cancer rates in Jamaican women were intermediate between the low levels in African countries and the high levels in USA, UK and other Western industrial societies.

Lastly, I am interested to know if data concerning the parity and age of first full-term pregnancy were recorded by Bowen *et al*. Pathak *et al* (2000) suggested that women of African-American, Hispanic, American-Indian and Hawaiian cultures present with more advanced stages of breast cancer due to earlier first full-term pregnancies and higher parities when compared to whites. So is this finding of Bowen *et al* due to ethnicity or confounding parity.

I think this study was worthwhile but the real question is how will these findings that black women get breast cancer at a younger age and are twice as likely to die of the disease than white women be incorporated into the UK breast-screening programme. There is also a need to study other ethnic groups because UK has a vast multi-cultural population and there are ethic variations with other types of carcinomas.
